# Vaccination against Q fever for biodefense and public health indications

**DOI:** 10.3389/fmicb.2014.00726

**Published:** 2014-12-16

**Authors:** Sara Ruiz, Daniel N. Wolfe

**Affiliations:** ^1^Center for Aerobiological Sciences, U.S. Army Medical Research Institute of Infectious DiseasesFort Detrick, MD USA; ^2^Chemical and Biological Technologies Department, Defense Threat Reduction AgencyFort Belvoir, VA USA

**Keywords:** Q fever, vaccine, biodefense, *Coxiella*, epidemiology

## Abstract

*Coxiella burnetii* is the etiological agent of Q fever, a disease that is often spread to humans via inhalational exposure to the bacteria from contaminated agricultural sources. Outbreaks have been observed all over the world with larger foci generating interest in vaccination programs, most notably in Australia and the Netherlands. Importantly, exposure rates among military personnel deployed to the Middle East can be relatively high as measured by seroconversion to *C. burnetii*-specific antibodies. Q fever has been of interest to the biodefense community over the years due to its low infectious dose and environmental stability. Recent advances in cell-free growth and genetics of *C. burnetii* also make this organism easier to culture and manipulate. While there is a vaccine that is licensed for use in Australia, the combination of biodefense- and public health-related issues associated with Q fever warrant the development of a safer and more effective vaccine against this disease.

## Q FEVER DISEASE

An unknown disease was reported in Queensland, Australia in 1933, termed Query Fever (Q fever) that caused non-descript symptoms including fever, headache, and general malaise. The etiological agent, *Coxiella burnetii*, was described in Davis (1938) and Waag and Fritz (2012). This gram-negative bacterium is an obligate, intracellular pathogen, oscillating between an infectious small cell variant and a replicative large cell variant (McCaul and Williams, 1981). *C. burnetii* is a zoonosis, persisting within domesticated ungulates, such as cattle, horses, sheep, and goats (Langley et al., 1988; Laughlin et al., 1991).

*Coxiella burnetii* exposure results from contaminated animal byproducts, with human exposure often occurring via inhalation (Lennette and Welsh, 1951). Infectious particles can travel several kilometers by wind leading to epidemics (Tissot-Dupont et al., 2004). Although uncommon relative to inhalational exposure, transmission of the bacteria can occur by ingestion of unpasteurized milk and vectors, specifically ticks (Davis, 1938; Huebner et al., 1948). The minimum inoculum of *C. burnetii* is estimated to be 1.18 bacteria with an estimated ID_50_ of 5.58 bacteria, underscoring the potential of this bacterium to cause a significant public health toll (Brooke et al., 2013).

Many exposed individuals remain asymptomatic, ∼60%; however, those that develop acute Q fever have no distinguishing clinical signs or symptoms and generally present with malaise, fever, headache, chills, and can progress to pneumonia. Acute hepatitis with an elevation of aspartate transaminase and/or alanine transaminase has also been reported (Palmela et al., 2012). Acute disease is typically self-limiting with low mortality (Waag and Fritz, 2012). Contraction of disease during pregnancy, however, can result in complications such as premature birth, stillbirth, and low birth weight due to bacterial tropism for the placenta (Ellis et al., 1983; Stein and Raoult, 1998; Jover-Diaz et al., 2001; Langley et al., 2003). All individuals who have been exposed to *C. burnetii* are at risk of developing chronic Q fever (Brooke et al., 2013, 2014), with an estimated 1–5% progressing to chronic Q fever, placing them at risk of serious long-term sequelae (Botelho-Nevers et al., 2007; Million et al., 2010).

Individuals with pre-existing cardiac valvular disease, aortic aneurysm, vascular grafts, immunocompromised status, and pregnancy at time of exposure are at an increased risk for developing chronic Q fever (Raoult et al., 2000; Fenollar et al., 2001; Landais et al., 2007), which most commonly results in endocarditis or hepatitis (Yebra et al., 1988). Chronic fatigue syndrome is commonly observed in the short term following diagnosis (Brooke et al., 2014). The disability adjusted life years burdens were estimated for both H1N1 influenza and Q fever during the recent Netherlands epidemic, with the burden due to chronic Q fever being estimated at 8–28 times more severe per case compared to H1N1 influenza (Brooke et al., 2014). This highlights the need for better diagnostics and medical countermeasures, particularly in cases of chronic Q fever.

## Q FEVER DIAGNOSTICS AND MEDICAL COUNTERMEASURES

The current standard for Q fever diagnosis is a commercially available indirect immunofluorescence assay. Cultivation of the organism is not recommended given its high infectivity and requirement of Biosafety Level 3 containment. The limited utility of diagnostic assays for Q fever is exacerbated by the non-specific disease symptoms and lack of clinical indicators to suggest Q fever early in the course of disease. Culture and serum based PCR are only positive in 50–60% of chronically infected individuals (Fenollar et al., 2004). Antibody responses to the Phase I and Phase II antigenic variants allow for the differentiation between acute and chronic phases of disease. Phase I *C. burnetii* possess full-length lipopolysaccharide (LPS) whereas Phase II variants begin to appear in the chronic phase with a truncated LPS lacking O antigen (Schramek and Mayer, 1982; van der Hoek et al., 2012). PCR-based approaches have been explored given that bacterial DNA can be detected prior to the antibody response, thereby curtailing the diagnostic delay. A positive PCR is indicative of infection, but a negative result is inconclusive (Fournier et al., 1998). The combination of non-descript symptoms and inefficient assays makes the diagnosis of Q fever a fairly daunting challenge.

Although acute Q fever is typically self-limiting, a 2 weeks course of doxycycline is recommended. Chronic Q fever requires a much more intensive antibiotic regimen consisting of 18–24 months of doxycycline and hydroxychloroquine to resolve the infection (Kersh, 2013). A definitive study on the use of prophylactic antibiotic treatment for preventing chronic Q fever has not been undertaken. Although it is suggested for individuals in high risk groups, the benefit and duration of treatment have not been described (Kampschreur et al., 2014).

Given the difficulties associated with the treatment of chronic Q fever, vaccinations have been considered a viable alternative for at-risk populations. The utilization of Q-Vax, a formalin-inactivated whole cell vaccine, in Australia is very promising demonstrating that effective, vaccine-mediated protection upward of 5 years against Q fever is possible. (Ackland et al., 1994). However, Q-Vax does pose issues for individuals who have been previously exposed to *C. burnetii*. Individuals must undergo serology and skin tests prior to vaccination and use is restricted to individuals testing negative for both due to adverse reactions that can occur in previously sensitized individuals (Smadel et al., 1948). More recent development efforts focused on chemical extraction methods seeking to retain immunogenicity while trying to dampen the adverse reactions. Chloroform-methanol extraction of the Henzerling strain was shown to elicit robust protection but retained the undesirable adverse reactions (Williams and Cantrell, 1982; Williams et al., 1986).

## PUBLIC HEALTH AND BIODEFENSE CONCERNS

Q fever is a disease of worldwide distribution, but the prevalence varies widely and significant outbreaks do occur on occasion. For example, a large outbreak occurred in the Netherlands from 2007 to 2010, with over 4,000 cases being documented (Fries et al., 1993). Seroepidemiology studies have been conducted around the world in both humans and agricultural animals. **Table 1** provides a synopsis of key studies with respect to the seroprevalence of *C. burnetii* (Marrie et al., 1984; Tellez et al., 1989; Letaief et al., 1995; Okabayashi et al., 1999; Ko et al., 2000; Kim et al., 2006; Kilic et al., 2008; McCaughey et al., 2008; Anderson et al., 2009; Gozalan et al., 2010; Frankel et al., 2011; Tozer et al., 2011; Esmaeili et al., 2014; Schimmer et al., 2014; van Wijk et al., 2014). This synopsis sought to focus on those studies that did not specifically target at-risk individuals such as agricultural workers. Most studies found seroprevalence rates between 3 and 15%. However, a few did suggest rates as high as 20–30%.

**Table 1:** Highlights of seroprevalence of Q fever by nation.

Nation	Seroprevalence (%)
Australia ( Tozer et al., 2011)	5.3^a^
Australia ( Tozer et al., 2011)	5.0^a^
Canada (Marrie et al., 1984)	4.1/11.8^b^
Canada (Marrie et al., 1984)	5.0/14.6^b^
France (Frankel et al., 2011)	2.1
Iran (Esmaeili et al., 2014)	27.8^c^
Netherlands (van Wijk et al., 2014)	4.4
Netherlands (Schimmer et al., 2014)	72.1^d^
Northern Ireland (McCaughey et al., 2008)	12.8
South Korea (Kim et al., 2006)	1.5
Spain (rural) ( Tellez et al., 1989)	15.4
Spain (urban) ( Tellez et al., 1989)	8.8
Taiwan (Ko et al., 2000)	4.2
Tunisia (Letaief et al., 1995)	26
Turkey (Gozalan et al., 2010)	13.5
Turkey (Kilic et al., 2008)	32.3
United States (Anderson et al., 2009)	3.1
Zambia (Okabayashi et al., 1999)	8.2

*^a^Comparisons were made between rural (5.3%) and urban (5.0%) populations*.

*^b^Percentages reflect seropositivity by complement fixation and microimmunofluorescence assays*.

*^c^Population was focused on hunters, farmers, health care workers and others who were referred by diagnostic laboratories*.

*^d^Population was focused on agricultural workers in close association with infected cattle*.

The endemnicity of Q fever in Australia has warranted the use of Q-Vax in vaccination programs. Research into Q fever in Australia has been at least in part driven by economic considerations. Analyses of Q fever from 1991 to 1994 suggested that the costs associated with the disease were over $1 million Australian dollars and 1,700 weeks of work annually across Australia (Garner et al., 1997). While relatively small compared to other diseases, the availability of a low cost, effective vaccine has provided an economic benefit.

Some of the highest rates of prevalence for Q fever have been reported in the Middle East and studies have been conducted in parallel in both human and animal populations. Recent surveys in Iran demonstrated high rates of seropositivity in both humans and sheep. An analysis of sheep showed that 23.7% of animals had antibodies against *C. burnetii* (Esmaeili et al., 2013) and parallel studies in human cohorts demonstrated a seropositivity rate of 27.8% which correlated with handling of agricultural animals (Esmaeili et al., 2014). Clinical investigation of febrile illnesses of unknown origin in Saudi Arabia showed that out of 51 patients who had febrile illness in the past 4–8 weeks, 35% were seropositive for Q fever compared to just 4% of the control subjects (Almogren et al., 2013). Similar analyses in an Iranian cohort demonstrated that 24 and 36% of febrile patients were positive for antibody responses against phase I and II antigens, respectively (Khalili et al., 2010), highlighting the endemnicity of Q fever in the Middle East.

Q fever is a concern for the military due to deployments to regions where the disease is present at relatively high levels. In an analysis of a group of U.S. troops that were deployed to an area of Iraq with known Q fever outbreaks, 7.2% seroconverted during the deployment (Royal et al., 2013). A larger study of deployed Warfighters followed a cohort of patients that were admitted to hospitals with fever and other non-specific symptoms. Out of 909 individuals, 88 were seropositive for Q fever (Anderson et al., 2011). An outbreak among a smaller group of US Marines that were deployed to Iraq resulted in 22/38 developing Q fever (Faix et al., 2008). Similar findings have been reported by the United Kingdom military as well. In a 6 months period from May to October, 2008, 26 cases of Helmand Fever were identified in British troops, six of which proved to be Q fever (Bailey et al., 2011). Thus, while the threat posed by Q fever in the United States is fairly low from a domestic public health perspective, the recent clinical serology data suggest that *C. burnetii* represents a substantial disease burden among the deployed military.

Medical countermeasures against Q fever are of interest from a biodefense perspective. The ease of aerosolization of *C. burnetii*, its low infectious dose, and environmental stability led to its use in offensive programs prior to the Biological Weapons Convention (Martin and Eitzen, 2007). The United States included *C. burnetii* testing in Operation Whitecoat, wherein conscientious objectors were intentionally exposed and monitored for signs of illness and promptly treated with oxytetracycline (Benenson and Tigertt, 1956). An intentional release was also mimicked by discharging the bacterial aerosol from 3000 feet, or approximately 900 meters (Bellamy and Freedman, 2001). All exposed individuals were treated with antibiotics and followed for development of chronic infection (Tigertt et al., 1961). Testing on *C. burnetii* was also ongoing within the Soviet Union’s biological offense program during 1920s through 1990s (Pittman et al., 2005). The ubiquitous nature of *C. burnetii* in the environment, as well as its history in offensive programs, have spurred interest in vaccine programs. To date, vaccine development efforts have been largely based in empirical studies, partially due to the difficulties associated with culturing and manipulating *C. burnetii*. However, there have been key advances in Q fever biology that can now greatly facilitate the rational design of vaccines.

## ADVANCES IN Q FEVER BIOLOGY

The ability to generate defined mutants of *C. burnetii* and grow the bacterium in cell-free media have provided two major tools that will continue to facilitate Q fever studies (van Schaik et al., 2013). The development of an axenic culture media that support extracellular growth has been crucial to recent advances. The first generation was a complex *Coxiella* medium that allowed protein and ATP synthesis upward of 24 h. Metabolism was retained in a citrate-based buffer supplemented with three complex nutrient sources; neopeptone, fetal bovine serum (FBS), and RPMI cell medium (Omsland et al., 2008). The second generation medium, Acidified Citrate Cysteine Medium demonstrated bacterial recovery on par with that of Vero cell growth upon the addition of L-cysteine (Omsland et al., 2009). The current axenic medium replaced FBS with methyl-β-cyclodextrin. This medium formulation has been used to successfully culture bacteria directly from infected animal tissue homogenate. In addition, the doubling time was decreased, transition between SCV and LCV during stationary phase has been observed, and by adding a soft agar layer facilitates the isolation of pure colonies. (Omsland et al., 2011; Sandoz et al., 2014). Vero cell extract medium was also found to be permissive to *C. burnetii* growth, with the bacteria retaining its antigenicity profile (Singh et al., 2013). Taken together, the advent of cell-free growth media has facilitated the development of new genetic tools to be tested in *C. burnetii* (van Schaik et al., 2013).

The ability to transform Phase II *C. burnetii* was demonstrated by conferring chloramphenicol resistance and mCherry red fluorescent protein by Himar 1 transposon (Beare et al., 2009). Twenty clonal isolates of Type IV Secretion System substrate mutants were identified using the same Himar 1 transposon and transposase approach. Screening of these isolates produced 10 that were defective in intracellular replication and vacuole formation, providing insight into the lack of effector redundancy as observed in *Legionella pneumophila* and potential novel virulence factors (Weber et al., 2013). Two novel gene deletion systems were developed in *C. burnetii*, a Cre-lox mediated recombination and the loop in-loop out strategy. Using the Cre-lox-mediated recombination, the structural component of type IVB secretion system, dotA, was deleted. A double mutant of dotA and dotB was created with the loop-in/loop-out method in which a suicide plasmid with sacB-mediated counterselection generated an unmarked mutation (Beare et al., 2012). Furthermore, saturation of the genome by RNA interference was recently used to create a library of mutants to allow for screening of essential host pathways for successful *C. burnetii* infection (McDonough et al., 2013). A greater understanding of *C. burnetii* pathogenesis has, and will continue to, drive investigations into the rational design of Q fever vaccines. Recent work has suggested that specific T cell epitopes may be necessary in the successful design of a next-generation vaccine against *C. burnetii* (Xiong et al., 2014).

## PROGRESS TOWARD A Q FEVER VACCINE

As noted above, Q-Vax has been licensed for use in Australia highlighting an important success by effectively reducing the disease burden as part of a preventative vaccine program. Q fever reporting declined by ∼50% from 2002 to 2006 upon the implementation of a vaccine program targeted toward abattoir workers and farmers (Gidding et al., 2009). Trials have also been conducted in at-risk older adults in the Netherlands. Although not as promising as immunogenicity data in younger cohorts, patients in this study did demonstrate positive responses by ELISA (46%) and IFN-γ assays (67%) after 6 months and 1 year (60% for both assays; Schoffelen et al., 2013). In a related study with a formalin-inactivated vaccine, 13 out of 16 individuals responded to the vaccine with positive measures of antibody titers (titer > 1:8) and IFN-γ responses (>31.1 pg of IFN-γ produced; Kersh et al., 2013).

Although the above trials have provided much hope in terms of an effective Q fever vaccine, there are some issues that remain to be addressed. Protection has clearly been shown in multiple efficacy trials and experiments and a meta-analysis of a range of vaccine campaigns with Henzerling phase I vaccines supported the conclusion that these vaccines do indeed protect against acute disease. However, there were differences when comparing individual studies (O’Neill et al., 2014). Perhaps more importantly, the various forms of inactivated *C. burnetii* vaccines require a pre-sensitivity screening via skin tests and serology. It is important to note that in a recent vaccine campaign in the Netherlands, 22% of individuals had to be excluded from the trial due to positive skin tests and/or serology (Isken et al., 2013). From the perspective of developing a vaccine for the biological defense of the U.S. military, the required pre-screening and inability to vaccinate pre-sensitized individuals poses a logistical challenge that warrants investment in a next-generation Q fever vaccine.

Since the development of Q-vax and other related whole-cell inactivated vaccines, the funding for Q fever vaccines, as well as *C. burnetii* in general, has been limited. In terms of next-generation vaccines, the candidate that has moved the furthest along the pathway toward licensure is a chloroform-methanol residue vaccine. This candidate was taken forward into a phase I clinical trial that demonstrated immunogenicity when administered with a primary vaccination and boost 3–6 months later (Waag et al., 2008). However, the priming doses did not induce an increase in *C. burnetii*-specific antibody titers and individuals with positive titers were excluded from the study (Waag et al., 2008), thus, it is not clear if the chloroform-methanol residue would have the same issue as inactivated vaccines with respect to pre-exposed individuals. The authors of this study discussed ongoing efforts to characterize this vaccine in individuals who were positive for reactions to Q fever via a skin test, but nothing has been published with respect to this to date.

Much of the more recent progress in Q fever has involved a more basic understanding of factors involved in protection against infection as opposed to vaccine development. Phase I LPS has been associated with protection against Q fever in multiple studies (Zhang et al., 2007; Peng et al., 2012). A Phase I LPS-specific monoclonal antibody was shown to be protective in a mouse model of infection (Peng et al., 2012). Utilization of a phage display library resulted in the discovery of a peptide mimetic that mimics a protective epitope of Phase I LPS and elicits a protective antibody response in mice (Peng et al., 2012). There has been somewhat limited progress toward the identification of protein antigens that may constitute viable antigens to be included in a next-generation Q fever vaccine. Com1 and Mip surface-exposed proteins have been shown to be immunogenic in human clinical samples (Vigil et al., 2011). Immunization of mice with dendritic cells stimulated with these proteins resulted in increased rates of bacterial clearance, suggesting that the immune response to these proteins may also contribute to protection against disease (Xiong et al., 2012). Stimulation of T cells with Com1-pulsed monocyte-derived dendritic cells resulted in proliferation and activation of T cells (Wang et al., 2011).

Additional insights have also been gained into the type of immune response that may mediate protection against *C. burnetii*. Protection has been associated with antibodies to LPS in past studies (Zhang et al., 2007; Peng et al., 2012), and it has been suggested that although antibodies do contribute to protection, Fc receptors and complement are not required for the mechanism of action (Shannon et al., 2009). There is also a growing appreciation of the role of T cells in protection against Q fever. Passive transfer of bone marrow-derived dendritic cells stimulated with *C. burnetii* antigens resulted in protection against Q fever. Stimulation with Phase II antigens elicited robust protection, but Com1 and SecB alone elicited partial protection that correlated with CD4^+^ T cell responses (Wei et al., 2011). Work in SCID mouse models have shown that CD4^+^ or CD8^+^ T cells were sufficient to provide protection against disease (Read et al., 2010). Vaccine studies utilizing formalin-inactivated *C. burnetii* have shown that T cells are particularly important in vaccine-mediated protection against Q fever (Zhang et al., 2007).

## FUTURE INVESTMENTS IN Q FEVER MEDICAL COUNTERMEASURES

While an effective vaccine is currently in use in Australia, a greater understanding of Q fever may enable the development of a next-generation vaccine with fewer reactogenicity issues. Relative to other Biodefense programs, there can be a much stronger tie between biodefense and public health indications.

Given the relative lack of information regarding protein antigens of *C. burnetii* that may constitute viable protective antigens to be included in vaccine formulation, further investments in antigen discovery for Q fever are warranted. The number of clinical Q fever cases around the world has potential to provide key insights into the discovery phase of a next-generation vaccine. For example, protein microarrays have been used to interrogate the antibody responses in Q fever patients, which covered approximately 93% of the *C. burnetii* proteome and have used this tool to study the immune responses to Q fever infection (Vigil et al., 2011). Clinical immunology studies would be of continued utility in understanding *C. burnetii* antigens that are immunogenic and may contribute to either protective immune responses or reactogenicity issues associated with vaccinations of previously exposed individuals with whole cell Q fever vaccines.

The ability to tease out protective immune responses versus those involved in reactogenicity will enable rationally designed Q fever vaccines that would not require the requisite pre-screening. In order to develop such a vaccine, the Q fever research community needs the tools to study the immune response to *C. burnetii*. Well-characterized assays will be needed to analyze the immune responses, most likely both antibody and T cell responses, to the antigens being included in next-generation vaccines. If a Q fever vaccine is to be licensed for use, regardless of pre-exposure status, care will need to be taken in animal model experimentation to assess the likelihood of pre-exposure sensitivity.

Concerted efforts to (1) identify optimal antigens to be included in a vaccine, (2) develop models pre-exposure sensitivity to Q fever vaccination, and (3) demonstrate proof-of-concept safety and efficacy against *C. burnetii* challenge could provide candidate countermeasures in the relatively near future. As the community drives toward late-stage vaccine candidates, there will be opportunities for partnerships between the biodefense, public health, and agricultural stakeholders in order to achieve a Q fever vaccine that is relevant to the needs of the broader community as well as the Warfighter.

## AUTHOR CONTRIBUTIONS

Sara Ruiz and Daniel N. Wolfe both contributed to the analysis of data in the literature, drafting and revising the manuscript, approving for publication and will be accountable for resolving any questions that may result from this work.

## Conflict of Interest Statement

The authors declare that the research was conducted in the absence of any commercial or financial relationships that could be construed as a potential conflict of interest.
